# Ablation of rat TRPV1-expressing Adelta/C-fibers with resiniferatoxin: analysis of withdrawal behaviors, recovery of function and molecular correlates

**DOI:** 10.1186/1744-8069-6-94

**Published:** 2010-12-17

**Authors:** Kendall Mitchell, Brian D Bates, Jason M Keller, Matthew Lopez, Lindsey Scholl, Julia Navarro, Nicholas Madian, Gal Haspel, Michael I Nemenov, Michael J Iadarola

**Affiliations:** 1 Neurobiology and Pain Therapeutics Section, Laboratory of Sensory Biology, National Institute of Dental and Craniofacial Research, NIH, Bethesda, MD, USA; 2 Department of Anesthesia, Stanford University, Palo Alto, CA, USA; 3 Lasmed LLC, 137 Irene Ct., Mountain View, CA, USA; 4 Laboratory of Neural Control, Section on Developmental Neurobiology, National Institute of Neurological Disorders and Stroke, NIH, Bethesda, MD, USA

## Abstract

**Background:**

Ablation of TRPV1-expressing nociceptive fibers with the potent capsaicin analog resiniferatoxin (RTX) results in long lasting pain relief. RTX is particularly adaptable to focal application, and the induced chemical axonopathy leads to analgesia with a duration that is influenced by dose, route of administration, and the rate of fiber regeneration. TRPV1 is expressed in a subpopulation of unmyelinated C- and lightly myelinated Adelta fibers that detect changes in skin temperature at low and high rates of noxious heating, respectively. Here we investigate fiber-type specific behaviors, their time course of recovery and molecular correlates of axon damage and nociception using infrared laser stimuli following an RTX-induced peripheral axonopathy.

**Results:**

RTX was injected into rat hind paws (mid-plantar) to produce thermal hypoalgesia. An infrared diode laser was used to stimulate Adelta fibers in the paw with a small-diameter (1.6 mm), high-energy, 100 msec pulse, or C-fibers with a wide-diameter (5 mm), long-duration, low-energy pulse. We monitored behavioral responses to indicate loss and regeneration of fibers. At the site of injection, responses to C-fiber stimuli were significantly attenuated for two weeks after 5 or 50 ng RTX. Responses to Adelta stimuli were significantly attenuated for two weeks at the highest intensity stimulus, and for 5 weeks to a less intense Adelta stimulus. Stimulation on the toe, a site distal to the injection, showed significant attenuation of Adelta responses for 7- 8 weeks after 5 ng, or 9-10 weeks after 50 ng RTX. In contrast, responses to C-fiber stimuli exhibited basically normal responses at 5 weeks after RTX. During the period of fiber loss and recovery, molecular markers for nerve regeneration (ATF3 and galanin) are upregulated in the dorsal root ganglia (DRG) when behavior is maximally attenuated, but markers of nociceptive activity (c-Fos in spinal cord and MCP-1 in DRG), although induced immediately after RTX treatment, returned to normal.

**Conclusion:**

Behavioral recovery following peripheral RTX treatment is linked to regeneration of TRPV1-expressing Adelta and C-fibers and sustained expression of molecular markers. Infrared laser stimulation is a potentially valuable tool for evaluating the behavioral role of Adelta fibers in pain and pain control.

## Background

TRPV1 is a sodium/calcium ion channel expressed in a subpopulation of DRG neurons that respond to noxious heat, endogenous algesic compounds, and the vanilloid agonist capsaicin [1-3]. Capsaicin responses are detected in subpopulations of unmyelinated C-fiber neurons and myelinated Aδ-fibers [4,5]. Electrophysiological studies with radiant heat have shown that thermal sensing C-fibers mediate responses to stimuli that heat the skin at low rates (≤ 0.9°C/sec) whereas Aδ-fibers mediate responses to stimuli that heat the skin at high rates (≥ 6.5°C/sec) [6]. TRPV1 in C-fibers is responsible for burning pain sensations plus the integration of inflammatory chemical signals in many pathological pain states, and multiple drug development efforts have been directed at antagonizing TRPV1 for pain control [7-9]. TRPV1 agonists, such as the ultrapotent capsaicin analogue resiniferatoxin (RTX), have also been proposed as therapeutic agents for treating acute and chronic pain [10,11]. The binding of RTX leads to a sustained influx of sodium and calcium through TRPV1 channels [12] leading to channel desensitization and/or the loss of TRPV1-expressing DRG neurons and/or their fibers and terminals via calcium-induced cytotoxicity [13,14]. Thus, although the mechanisms diverge, ultimately either agonists or antagonists can be used as analgesic agents.

While antagonists can be administered orally, for agonists local administration is required and the route or site of administration is a critical factor. For example, RTX injection into the intrathecal space results in a loss of centrally projecting TRPV1-expressing fibers in the dorsal roots and at higher concentrations a loss of TRPV1-expressing DRG neuronal perikarya; both cases produce permanent regional analgesia [14-16]. In contrast, injections of low concentrations of RTX into peripheral sites (e.g., subcutaneous injections) spare the neuronal perikarya while ablating or temporarily inactivating TRPV1-expressing peripheral terminals and fibers [17,18]. This approach therefore results in temporary analgesia at focal sites until the fibers reactivate or regenerate. Systemic injections of RTX have also been used to induce analgesia; however, higher concentrations of RTX are needed and the analgesic effect is widespread rather than regional or focal [19].

Although TRPV1 is expressed in C- and Aδ-fibers, most animal studies that have ablated TRPV1 fibers with capsaicin or RTX focus on behavioral responses associated with C-fibers [14,17,20,21]. This can be attributed, primarily, to the prevalence of thermal pain assays that examine C-fibers rather than Aδ-fibers in awake rodents. Indeed, in the rat, where the axons are relatively short (20 cm or less), it may be difficult to distinguish behavioral responses mediated solely by fast-conducting Aδ-fibers. For example, the conduction velocity of the relatively slower C-fibers is sufficient enough to produce laser evoked potentials (LEP) as early as 225 ms after heating the paw with a brief CO_2 _laser pulse [22].

One goal of this study was to evaluate the usage of an infrared diode laser to examine behavioral responses that are discretely associated with Aδ activation in the rat. Infrared diode lasers, in contrast to CO_2 _lasers, are capable of directly heating the skin at depths where cutaneous nerve fibers terminate, thereby providing rapid (capable of > 200°C/sec), efficient, non-damaging thermal stimulation of Aδ-fibers [23-25], which can also initiate rapid behavioral responses. We combined RTX-induced ablation of TRPV1 fibers in plantar hind paw [17] with infrared laser stimulation and provide an excellent model to examine the contribution of Aδ-fibers to nociceptive behaviors in rats. Our findings demonstrate that RTX-induced thermal hypoalgesia is sustained longer than previously thought, and RTX greatly attenuates behavioral responses characteristic of Aδ stimulation. Importantly, this methodology suggests infrared laser stimuli can be used to analyze behavior associated with fast-conducting Aδ-fibers, which has been relatively difficult to accomplish in awake, unrestrained rodents.

## Results

We sought to specifically stimulate afferent nerve fiber subtypes in the glabrous skin of the rat hind paw using either short-pulse, high-rate (Aδ) or long-pulse, low-rate (C) skin heating with an infrared diode laser. Studies in rats have suggested that withdrawal activity following short-duration, high-intensity stimulation of the hind paw with a CO_2 _laser is mediated by C-rather than Aδ-fibers, in part from recordings of electromyographic (EMG) activity in the stimulated limb [26] and/or of laser-evoked cortical potentials (LEPs) [22,27,28], that show latencies consistent with a conduction velocity in the C-fiber range. The infrared diode laser used here has the ability to specifically activate Aδ fibers, which has been determined from direct extracellular recordings of trigeminal ganglion neurons in rats [25]. In order to further establish a relationship between Aδ conduction and nociceptive response a high-speed video camera (500 fps) was used to record stimulation-induced movements in awake, freely-moving rats during the brief peristimulus time period.

**Figure 1:**
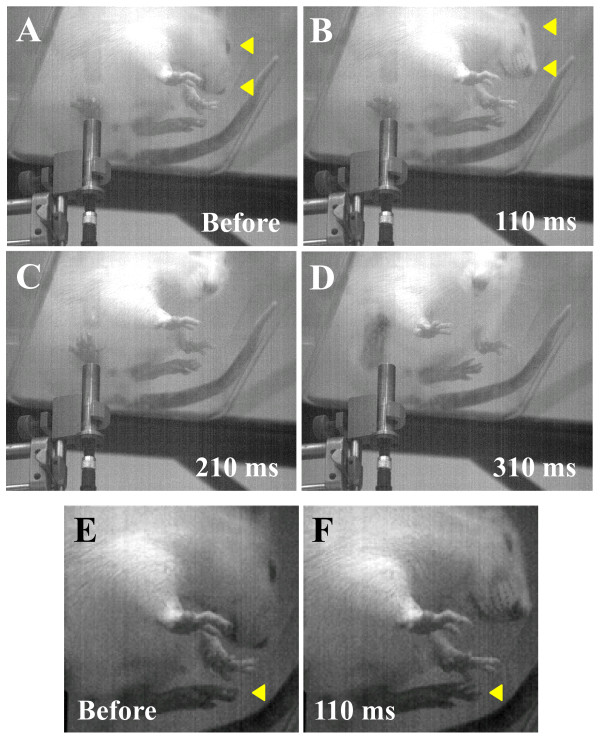
**Detection of early-onset nocifensive behaviors prior to withdrawal of stimulated limb**. A 100 msec thermal pulse at high power (6.08 W/mm^2^) was used to stimulate the right index toe. Withdrawal responses were recorded at a rate of 500 frames per second. The position of the rat's head before and after stimulation are highlighted in **A **and **B**, respectively. The upper and lower arrowheads in each frame bring attention to the eyes and nose, respectively. Abrupt head displacement in (**A**) versus (**B**) occurs by 110 msec (140-30 msec, see Methods). Also note in **B **the orbital "tightening", which was observed only with some rats following laser stimulation. (**C**) By 210 msec, the head was oriented towards the stimulated foot and toes on the stimulated foot have moved. (**D**) Withdrawal of the stimulated paw was underway by 310 msec. (**E**) and (**F**) are magnifications of (**A**) and (**B**), respectively, showing that by 110 msec, toe movement on the non-stimulated hind paw has preceded any movement detected in the stimulated limb. Full videos can be viewed in the Additional file 1 Movie S1 data and show that latency to the first detectable movement was approximately 74 msec.

With the high-speed camera we observed that the actual withdrawal of the stimulated limb was often preceded by a panoply of dedicated motor programs. For example, video frames of a rat before, and in the first 300 msec after stimulation (6.08 W/mm2) are shown in Figure 1. In this example, visible head movement towards the stimulated hind paw could be detected by 110 msec. The right eye was also seen to squeeze shut by 110 msec, however, this behavior was not a prominent feature in many of the trials. Figure 1C illustrates that by 210 ms, the stimulated toe has been retracted off the glass platform, and by 310 ms withdrawal of the hind limb is fully apparent (Figure 1D). The latency from the noxious phase (the period after the first 30 ms from when the laser stimulation began, see Methods) of the stimulus to the first observable laser-evoked movement (fLEM) in this trial was 74 ms which occurred in a forelimb rather than the stimulated limb (see also Additional file 1 Movie S1). In contrast, the latency to fLEM in the stimulated limb was 112 msec. Nerve length from the toe to the dorsal root entry zone was 16.8 cm, based on dissection (350 g rat). Hence, this example suggests a minimum conduction velocity of 2.27 m/s (0.168 m/0.074 s), which is well within the range for Aδ-fibers [≥ 1.3 m/s] [29,30] and beyond the range for the TRPV1 population of C-fibers [~0.4 m/s] [31].

Calculated estimates of conduction velocity based on latency to fLEM for a series of normal rats are listed in Table 1. We observed that response latency appeared highly dependent on the rat's posture at the moment of stimulation. From 16 video recorded trials, 6 of the 8 shortest latencies (n = 4 rats) to fLEM occurred when body weight was *not *supported by all four limbs. In these cases, one or both forepaws were not on the floor and hence the rat was in the "reared" position prior to stimulation. For these 16 trials, the shortest latency to fLEM was 56 ms and the average was 159.5 ± 36.7 msec. In contrast, 6 of the 8 longest latencies to fLEM occurred when weight was distributed to all four paws; from these trials, the shortest latency was 74 ms and the average was 250.0 ± 28.9 msec. It should be emphasized that our conduction velocity estimates are conservative. They do not account for central delay (possibly as high as 80 ms in the rat) [32], motoneuron conduction velocity (33-85 m/s) [33] or the time required for muscle contraction/relaxation (34-116 ms) [33], which would necessarily yield faster conduction velocity estimates. Furthermore, the cascade of bilateral extensor/flexor muscle activity required for abrupt postural adjustment is complex, and the possibility is strong that the earliest muscle twitches in some trials may have been missed entirely by the viewing angle of the camera. We cannot predict, at present, where movement will occur first. Despite this, we could capture in 31% of trials movements associated with nerve conduction velocities higher than 1.7 m/s, clearly within the range for Aδ-fibers, following a short, intense noxious laser stimulus. We conclude that Aδ-fibers must be recruited in some, if not all, responses under this stimulus paradigm.

**Table 1:** Latency to observable movement following short-pulse (100 ms), high intensity stimulation with infrared diode laser (6.08 W/mm2)

	Standing on 2 or 3 paws	Standing on 4 paws	Total
Total number of trials	8	8	16
Shortest latency to fLEM	56 ms	74 ms	56 ms
†Fastest conduction velocity	3.0 m/s	2.3 m/s	3.0 m/s
Mean latency to fLEM (± SEM)	159.5 ± 36.7 ms	250.0 ± 28.9 ms	204.8 ± 25.4
†Mean conduction velocity	1.1 m/s	0.7 m/s	0.8 m/s
Shortest latency to withdrawal of stimulated limb	74 ms	128 ms	74 ms
Mean latency to withdrawal of stimulated limb (± SEM)	213.0 ± 34.1	271.0 ± 27.7	242.0 ± 22.5

†Conduction velocities are likely a gross underestimation of the actual conduction velocities. They do not account for central delay (~80 ms in the rat) [32], motoneuron conduction velocity (33-85 m/s) [33] or the time required for muscle contraction/relaxation (34-116 ms) [33]. Nonetheless, we were able to demonstrate that it is possible to detect nocifensive behaviors that are temporally consistent with being mediated by Aδ-fibers. The posture of the animal before stimulation appears to greatly influence our ability to detect movements with short or longer latencies.

**Figure 2:**
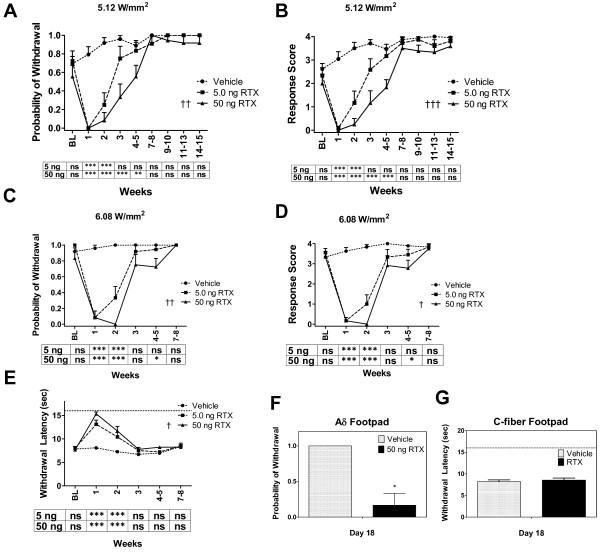
**Effect of RTX on Aδ and C responses at the mid-plantar injection site**. Either 5 or 50 ng of RTX was injected into the right while vehicle was injected into the left hind paw. Aδ and C responses before and after injections were followed for several weeks. (**A-D and F**) Aδ responses following intraplantar RTX-injection. We used probability of withdrawal (**A**, **C and F**) and the intensity of withdrawal (**B **and **D**) as endpoints for the Aδ assay. Behavioral responses to stimulus intensities at 5.12 W/mm^2 ^(**A**, **B **and **F**) or 6.08 W/mm^2 ^(**C **and **D**) are shown. (**E**) Loss and return of C-fiber responses after RTX-intraplantar injection. We used latency to paw withdrawal as the endpoint for the C-fiber assay; a stimulus intensity was chosen that produced a response latency of ~8 sec in normal rats. A cutoff of 16 sec (dashed line) was imposed to prevent tissue damage. (**F and G**) Direct comparison of behavioral responses at day 18 post-RTX to Aδ (**F**) and C (**G**) stimuli. Data used in week 3, in the line graphs, are the average of tests done on days 16, 18 and 21. Hypoalgesia at day 18 was maintained in Aδ but not C-fibers. The tables under the graphs denote statistical comparisons between vehicle and 5 ng or 50 ng RTX, which are aligned with the post-treatment time points. Each data point is an average of the combined responses from 2 or 3 non-consecutive days of testing during the time period indicated on the x-axis (weeks). For graphical simplicity, the data from all vehicle-treated rats (n = 12) is combined, yielding a single line in the plots, since there was no significant difference between the two vehicle groups. 2-way ANOVA with repeated measures was used to compare the effect of 5 ng or 50 ng RTX versus vehicle. 2-way ANOVA was used to compare the effects of 5 ng versus 50 ng. Fisher's exact test was used in **F**. *,† p < 0.5, **,†† p < 0.01, ***, ††† p < 0.001, n = 6 rats per group.

Rats were treated with intraplantar injection of 5 or 50 ng RTX and examined behaviorally over several weeks. Two stimulus intensities were used to examine the effect of RTX on Aδ-fiber activity. At baseline, the probability of withdrawal to the lower power stimulus (5.12 W/mm^2^) was 0.69 ± 0.08 and the severity of withdrawal (on the behavioral rating scale, see Methods) was 2.61 ± 0.24 (Figure 2A and 2B). After RTX injection, the probability of withdrawal and the severity of withdrawal were eliminated or barely detectable during the first two weeks. The 50 ng RTX-treated paws remained significantly suppressed for up to 5 weeks. By week 7, there was no longer a significant difference in behavioral responses to the Aδ stimulus between the RTX and vehicle treated paws. A statistically significant difference was observed between the 5 and 50 ng doses over the course of the study (p < 0.01, probability of withdrawal, Figure 2A; p < 0.001, intensity of withdrawal, Figure 2B).

When stimulating with higher power (6.08 W/mm^2^), the probability of withdrawal and intensity of withdrawal were close to maximum at baseline (0.92 ± 0.05 and 3.33 ± 0.19, Figure 2C and 2D, respectively). The stronger behavioral responses with the 6.08 W/mm^2 ^stimulus indicate more efficient heating of the skin as compared to the 5.12 W/mm^2 ^stimulus. After 50 ng RTX, rats were almost completely unresponsive to the 6.08 W/mm^2 ^Aδ stimulus for the first two weeks. By three weeks there was a substantial return towards baseline, nonetheless, a small but significant difference could be detected up to week 5 suggesting a small deficit in fiber function remained. By week 7, there was no difference between vehicle and RTX-treated paws, similar to the 5.12 W/mm^2 ^setting. Responses from the 5 ng RTX treated paws returned to baseline levels by week 3. As with the lower power setting, a statistical difference between the 5 and 50 ng groups was observed when stimulating with the 6.08 W/mm^2 ^laser pulse (p < 0.01, probability of withdrawal, Figure 2C; p < 0.05, intensity of withdrawal, Figure 2D).

Our previous work demonstrated that 62.5 ng of RTX attenuates C-fiber responses for about 2 weeks [17]. In the present study, normal rats withdrew from the laser generated C-fiber stimulus at about 8 s (Figure 2E). In rats lightly anesthetized with 0.5% isoflurane, a withdrawal response is retained, and the skin temperature (stimulation site) at the time of withdrawal was 48.5 ± 0.5°C when measured using a thermal camera [data not shown]. Both doses of RTX produced significant increases in withdrawal latency compared to the vehicle injected (contralateral) paw. Withdrawal latencies at the mid-plantar injected site reached cutoff (16 sec) during week 1, the latencies then decreased by week 2 but remained significantly different from contralateral paws until they finally normalized by week 3 (Figure 2E). Analgesia occurred with both doses of RTX and the time course of recovery was similar but the effect, however, was greater in the 50 ng group (p < 0.05). Figure 2F and 2G directly compare behavioral responses to the Aδ and C stimuli on day 18; after 50 ng RTX, rats were generally unresponsive to the 5.12 W/mm^2 ^Aδ stimulus (as compared to vehicle (p < 0.05). In contrast, responses to the C stimulus were not significantly different between the RTX and vehicle groups by day 18.

**Figure 3:**
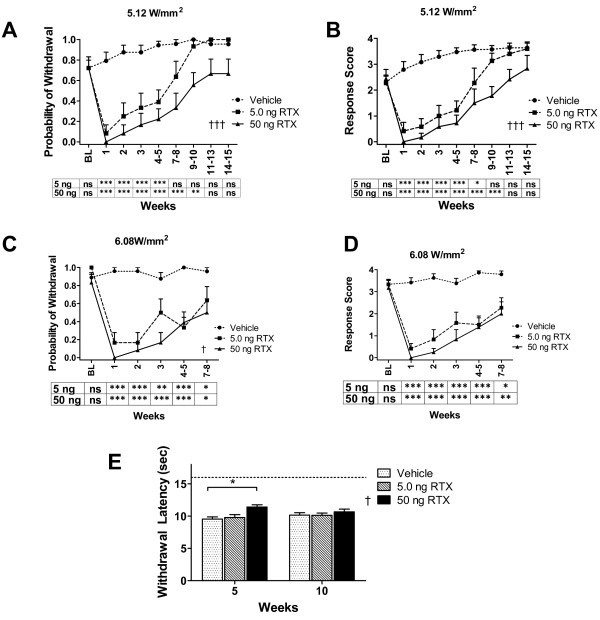
**Aδ- and C-fiber responses in the toes after mid-plantar RTX injection**. The infrared diode laser was used to stimulate Aδ- or C-fibers in the toes of the same rats used in Figure 2. (**A**-**D**) Responses to Aδ stimuli are diminished for up to 10 weeks. Plots are similar to those in Figure 2. (**E**) For 50 ng RTX only, a small but significant difference was measured in the toes at 5 weeks (but not at 10 weeks) for C responses; data reported are an average of 3 test days. Our initial protocol used C-fiber testing only at the mid-plantar footpad; however, since we observed prolonged, diminished Aδ behavioral responses in toes after RTX-treatment, a suppression of C-fiber behavioral responses might occur in tandem. During a separate study, we found that within 1 week after 50 ng intraplantar RTX, rats reached the 16 sec cutoff when testing C-fibers in their toes. 2-way ANOVA with repeated measures was used to compare the effect of 5 ng or 50 ng RTX versus vehicle. 2-way ANOVA was used to compare the effects of 5 ng versus 50 ng. *,† p < 0.5, **,†† p < 0.01, ***,††† p < 0.001, n = 6 rats per group.

The mid-plantar hind paw is innervated by the tibial nerve. Since the toes are also innervated by the tibial nerve, we separately examined the effect of intraplantar RTX on recovery kinetics in the toes. The probability of withdrawal for the toes using the 5.12 W/mm^2 ^Aδ stimulus was 0.72 ± 0.08 prior to RTX or vehicle treatment, similar to that obtained with mid-plantar stimulation. By 4 weeks, the probability of withdrawal and the intensity rating were near maximal for the vehicle-injected paw (0.94 ± 0.04 and 3.47 ± 0.16, respectively) (Figure 3A and 3B). In contrast, on the RTX injected side, the toe response was significantly reduced for at least 7 weeks for the 5 ng group and 10 weeks for the 50 ng group. Stimulation of the toes at higher intensity (6.08 W/mm^2^) resulted in higher baseline responses (probability of withdrawal = 0.89 ± 0.05 and response rating = 3.33 ± 0.21). Despite the stronger intensity, significant reductions in behavioral responses mediated by this Aδ stimulus could be seen for up to 7 weeks after intraplantar RTX (Figure 3C and 3D). These data show that behavioral sensitivity to Aδ stimuli is regained more slowly in the toes compared to the mid-plantar injection site. However, as with the mid-plantar site, analgesia in the toe was significantly greater in the 50 ng as compared to the 5 ng group (p < 0.001, Figure 3A and 3B; p < 0.05, Figure 3C).

Responses to C stimuli at week 5 revealed a small but significant difference between RTX (50 ng) and vehicle-treated toes (Figure 3E). By 10 weeks, there was no difference. There was no measurable statistical difference between the 5 ng RTX and vehicle-treated paws at week 5 or week 10.

**Figure 4:**
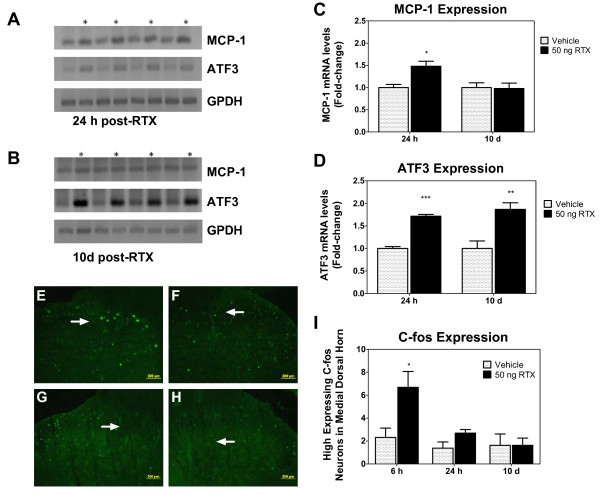
**Intraplantar injection of RTX leads to a transient increase in gene markers of nociception but a sustained increase in markers of nerve regeneration**. Gel image shows ganglionic expression levels of mRNA encoding MCP-1 and ATF3 24 h or 10 days after vehicle or RTX treatment, taken from left and right L4-L5 dorsal root ganglia (n = 4 rats, ipsilateral RTX expression is denoted by an asterisk). MCP-1 (**C**) and ATF3 (**D**) transcript levels were normalized to GPDH. Data were obtained from RT-PCR analysis (n = 4/group). All graphs are presented as mean ± SEM. *p < 0.05 and **p < 0.01, and ***p < 0.001 as determined by a one-way ANOVA followed by a Bonferroni correction. (**E**-**H**) Immunohistochemistry showing spinal cord c-Fos expression 6 h or 10 d post-RTX. **E **and **F **are epifluorescent images depicting an elevation in c-Fos protein 6 h after RTX or vehicle in the ipsilateral and contralateral dorsal horn, respectively. **G **and **H **are epifluorescent images representing c-Fos expression 10 d after RTX or vehicle in the ipsilateral and contralateral dorsal horn, respectively. (**I**) Cell counts demonstrate significantly more c-Fos immunopositive cells in spinal cord ipsilateral to RTX injection as compared to contralateral vehicle injections at 6 h but not at 24 h or 10 days. Data expressed as means ± SEM (n = 4). The fold-change is shown. *p < 0.05 as determined by Student's *t*-test. Arrows indicate the location of the medial dorsal horn. The most intense c-Fos positive neurons were counted in this area.

Application of RTX initially evokes nociceptive activity by binding to and activating TRPV1 [14]. This is followed by a dying-back (axonopathy) of unmyelinated and myelinated TRPV1-expressing fibers and potentially a recruitment of macrophages that can release cytokines, which in turn could activate the remaining proximal end of the nerve fiber. Using RT-PCR, we examined the DRG and spinal cord for expression of genes associated with nociceptive activity and nerve regeneration after RTX-treatment. As shown in Figure 4 the gene encoding the nociceptive chemokine MCP-1 [34,35] was increased in the DRG at 24 h, likely due to an initial burst of nociceptive activity following TRPV1 activation [36], but expression normalized by day 10 (Figure 4A-C). In contrast, the nerve regeneration marker ATF3 [37] was induced at 24 h but the increase was maintained through day 10 (Figure 4A, B and 4D). The regeneration marker galanin was also increased at day 5 and 10 (Additional file 2). Upregulated c-Fos in the spinal cord is also an early marker of nociception [38,39]. Consistent with the elevation in ganglionic MCP-1 transcripts, c-Fos protein levels increased early (6 h) but reverted by 24 h (Figure 4E-I), indicating that regeneration after RTX-induced axonopathy is devoid of ongoing spontaneous nociceptive activity sufficient to induce these markers.

## Discussion

In the present study, we examined (a) behavioral responses associated with thermal laser stimulation of Aδ- and C-fibers in awake rats, (b) such behavioral responses after peripheral ablation of the two fiber types following intraplantar administration of the vanilloid agonist RTX, (c) the time-course of recovery/regeneration, and (d) the expression of markers for nociception and nerve regeneration. The results demonstrate that the infrared diode laser can be tuned to evoke behavioral responses temporally consistent with activation of Aδ-fibers in the rat hind paw. The results also demonstrate that mid-plantar injection of RTX removes both Aδ- and C-fiber mediated thermal sensations in the footpad and toes. This is followed by a progressive recovery of behavioral function from proximal (mid-plantar) to distal (toes) over several weeks.

One of the main aims of the present report was to evaluate infrared laser evoked Aδ- and C-fiber nocifensive behaviors. Two approaches were used: analysis of behavioral reactions in the immediate 300 msec of the post-stimulus period and modulation of behaviors following intraplantar injection of RTX. We first established, with high-speed videography, that nocifensive behaviors following short duration (100 msec), high-intensity laser pulse stimulation (6.08 W/mm^2^) are mediated by Aδ-fibers, consistent with reported electrophysiological [24,25] and laser-evoked potential [22,26,40,41] observations. Importantly, the latency to withdrawal of the *stimulated *limb was not always useful for estimating the conduction velocities of activated fibers. Instead, by capturing the behavior of the entire animal with a high-speed camera, we often observed that a rapid set of movements occurs *before *withdrawal of the stimulated limb. In most cases the latencies of these early movements indicated a faster afferent conduction velocity than if the latency to withdrawal of the stimulated limb was used as the endpoint. The progression of motion after the first observable laser-evoked movement (fLEM) was often consistent with postural adjustments to ensure stability that preceded a later, yet brisk, withdrawal of the stimulated limb. The shortest latency to fLEM was 56 ms, yielding a conduction velocity estimate for the primary afferent volley of 3 m/s, which is in the range for Aδ fibers and well above the range for C-fibers. In a previous study using a CO_2 _laser, EMG recordings taken from the gluteus maximus and biceps femoris of the stimulated limb yielded evoked electrical activity at ~280 ms [26]. However, these studies were performed on anesthetized animals whereas the present observations, obtained from awake unrestrained rats, suggest that complex postural changes and even reflexive Aδ-type behaviors are suppressed when the animal is under anesthesia. Our high speed imaging of the earliest, reflex-like movements is indicative of an Aδ-mediated nociceptive response to 'first pain' [42] and an activation of the Aδ "alarm system" [43].

When rats supported their body weight on 2 or 3 paws, fLEM often occurred at < 160 ms and frequently the movement occurred on the contralateral (i.e., unstimulated) side. This suggests that a readily observable short latency Aδ-evoked movement is more likely to be revealed when rats are in a "reared" position, or any posture that opportunistically requires rapid mediolateral balance adjustment prior to paw withdrawal. In contrast, when rats are standing with body weight distributed to all four legs, and therefore more neutrally balanced, latency to fLEM increased to ~250 msec. When all four paws are on the ground, early postural adjustments are difficult to detect if they primarily involve the axial musculature rather than repositioning of the forepaws or head and neck, all of which can occur in the first 100 msec following the end of the laser pulse. An additional consideration is that in the rat, central delay to acute laser stimulation has been reported to last for up to 80 ms [32]. Whether this makes a significant contribution to "preparatory" adjustments [44] or long latency movements is being further investigated but it is clear that controlled balance at the moment of retraction of the stimulated paw is a priority for spinal sensory-motor circuits (for an example in humans, see [45]).

The actions of RTX are manifested in both Aδ- and C-fiber types. We observe that peripherally administered RTX temporarily eliminates behavioral responses to both Aδ- and C-fiber mediated thermal nociception. Our data corroborate previous human studies in which, after prolonged capsaicin treatment, cortical LEPs corresponding to activated Aδ- and C-fibers are eliminated, as well as the behavioral response [46,47]. The capacity to independently test the two fiber types using our current laser settings suggest differential recovery rate for Aδ compared to C-fibers. After administering the higher dose of RTX (50 ng), responses to the C-fiber stimulus recovered first, but responses to the Aδ stimulus lagged by several weeks. However, the C-fiber stimuli also covered a greater area of skin, increasing the likelihood of activating a functional nerve ending. In both cases, responses to the C-fiber and Aδ stimuli, which were initially eliminated, returned gradually rather than abruptly. The gradual return is consistent with the idea that, within a single fiber type, the rate of regeneration is heterogeneous. It is also possible that, during recovery, C-fibers played a more pronounced role in mediating behavioral responses as they were no longer preempted by the faster Aδ input [48]. The lag in recovery of the full, archetypal Aδ behavioral responses after RTX may indicate that the Aδ-fibers are greatly compromised and/or undergo a more extensive axonopathy and/or that return of function depends on complete re-myelination. Because the diameter of the Aδ laser spot was small we could stimulate multiple discrete areas of skin and spatially map the thermal sensitivity of the paw. Spatial mapping of the paw demonstrated a slower return of distal sensitivity (toes) compared to return of sensitivity proximal to the injection site (mid-plantar). Therapeutically, prolonged behavioral recovery at sites distal to an RTX injection indicates that regional analgesia can be sustained longer by injecting RTX more proximal to the DRG and has potential therapeutic ramifications for treatment of peripheral neuropathic pain problems subsequent to nerve injury or possibly chemotherapy.

During the first 3 to 5 weeks of repeated testing, we observed that the rats became progressively more sensitive to the Aδ stimulus (Figure 2 and 3). This may be due to one or more concurrent processes, including (a) cued or non-cued learning or anticipation, which may be greatly influenced by the aversive nature of the stimulus, and (b) habituation to the novelty of the testing environment. Interestingly, the rats did not become more sensitive to C-fiber stimuli over the course of the study. This likely reflects a specific difference between the C and Aδ stimuli. Our C-fiber test involves slow-rate heating over several seconds (average latency ~8 sec) and the sensation of warming occurs well before the stimulus becomes noxious. Even if distracted by environmental cues, the warming phase can act as a thermal cue for the subsequent noxious threshold. Nonetheless, the rats maintained consistent withdrawal latency to C-fiber stimuli and did not exhibit a trend towards shorter latencies after repeated testing. This suggests that neither learning or anticipatory cues nor habituation critically affect responses in longitudinal C-fiber testing. In contrast, the sharp, pricking sensation of an Aδ stimulus (as reported by human subjects, [49]) may make it substantially more aversive than a C-fiber stimulus and therefore couple it more tightly to anticipatory or learning circuits and suggest that further exploration of these aspects is warranted.

The initial RTX-induced nociceptive phase, subsequent analgesia, and nerve regeneration were each discernible at the molecular level. We assessed the temporal regulation of several molecular markers of nociceptive and regenerative processes in DRG or spinal cord following intraplantar injection of RTX. MCP-1 is widely thought to be a marker of pain since transcript and protein levels are upregulated in the DRG by peripheral inflammation [36] and in neuropathic pain models [50-52]. With intraplantar RTX, we observed that ganglionic MCP-1 was induced at 24 h, consistent with our previous findings [36]. The increase in MCP-1 was not sustained: no elevation was detected at day 5 (data not shown) or at day 10. In contrast, ATF3, a marker of nerve regeneration [37,53], exhibited an increase at 24 h that was sustained at both day 5 and 10, indicative of a long-lasting regenerative process after intraplantar injection of RTX. Strong upregulation of galanin, another marker of nerve regeneration, was also detected on day 5 and 10. Cumulatively these data suggest that processes involved in peripheral nerve regeneration do not contribute to the maintenance of MCP-1 upregulation. Further investigation may provide clues to the mechanisms of MCP-1 upregulation in neuropathic and inflammatory models of pain and the heterogeneity of neuronal expression in the DRG. At least in the case of RTX, action potentials or calcium signaling [12] may be involved, since RTX induces acute, prolonged nociceptive behaviors at the 50 ng dose [36] and a massive influx of calcium [13] precedes desensitization. It is also possible, however, that MCP-1 is induced by the initial dying back of the TRPV1-expressing fibers (e.g., due to a loss of trophic factor signaling) rather than the initial burst of nociceptive activity. Consistent with the transient increase in MCP-1 following peripheral RTX, we observed that expression of the c-*fos *gene, measured by c-Fos protein immunostaining, was transiently increased in the spinal cord immediately after intraplantar RTX injection, consistent with our previous findings [17]. Induction of c-Fos in the spinal cord is widely accepted as a marker of pain signaling [38,39]. A drop in c-*fos *gene expression towards baseline levels by 24 h further indicates that RTX induces an immediate inactivation of nerve fibers since sustained nociceptive input can produce a long-lasting change in Fos proteins in dorsal spinal cord [38]. The lack of sustained elevation in protein occurs concurrently with a pronounced behavioral analgesia.

## Conclusion

Together our data demonstrate that analgesia following intraplantar administered RTX lasts much longer than previously assumed. The previous underestimate is due, in part, to the undetected extended time required for complete behavioral recovery to an Aδ stimulus. Highspeed videography suggests that Aδ nociception engages a complex process of rapid sensory-motor integration involving coordinated postural and balance adjustments prior to limb retraction. Infrared laser stimulation in awake, unrestrained animals can be used to assess the behavioral role of Aδ-fibers in several pain models and to differentiate mechanisms related to myelinated and non-myelinated fiber populations.

## Methods

### Animals

Male Sprague-Dawley rats (250-350 g) were housed under a 12 hr light-dark cycle and allowed access to food and water ad libitum. The ambient temperature of the holding and testing rooms was 21-22°C. Procedures were performed in accordance with the National Institutes of Health (NIH) Guidelines for the Care and Use of Laboratory Animals, and approved by the National Institute of Dental and Craniofacial Research (NIDCR) Animal Care and Use Committee. All efforts were made to minimize both animal numbers and distress within the experiments.

### RTX administration

Sealed glass ampoules of RTX (1 mg) were obtained from LC Laboratories (Woburn, MA). RTX was solubilized in 150 μl ice-cold 100% ethanol, diluted with 50 μl ddH_2_O supplemented with ascorbic acid (to make a final RTX stock concentration of 5 μg/μl RTX and 2 mM ascorbic acid), and further diluted to a working concentration of 100 ng/μl in sterile vehicle (0.25% Tween-80, 2 mM ascorbic acid, 0.9% NaCl). Vehicle was also used to dilute RTX (100 ng/μl) to lower working concentrations. All intraplantar injections were made using a total volume of 50 μl.

### Thermal stimulus paradigm

An infrared diode laser (LASS-10 M; Lasmed, LLC, Mountain View, CA) with an output wavelength of 980 nm and maximum power of 20 W was used to generate thermal stimuli. For calibration, laser power/energy was measured using a meter with a thermal sensor (Nova II, L30A-10 MM, Ophir Optronics). Cutaneous C-fibers were selectively activated by low-rate heating using long pulses, low energy and a large diameter beam (5 mm Ø, nominal) [24]. Aδ-fibers were selectively activated with a high rate of heating, using a high-energy, brief pulse (100 ms), and a small spot size (1.6 mm Ø, nominal). A thermal damage cutoff for each stimulus paradigm was determined primarily by visual examination (e.g., presence of a spot of protein coagulation, acute redness or blistering, swelling 24 h post) of stimulated, anesthetized rats. We observed that Aδ pulses at 7000 mA (corresponding to a power density of 6.93 W/mm^2^) often resulted in instantaneous skin damage whereas 6000 mA (6.08 W/mm^2^), which evokes strong behavioral withdrawal responses, never produced visible skin damage. The skin heating rate for each Aδ stimulus was measured with an infrared camera (ThermoVision SC6000, FLIR Systems, Inc.) on the plantar foot pad of anesthetized rats, acquired at 400 fps. At 5000 mA (5.12 W/mm^2^) the rate was ~235°C/sec; at 6000 mA (6.08 W/mm^2^) the rate was ~300°C/sec. For the C-fiber stimulus (1000 mA; 0.083 W/mm^2^), a 16 sec cutoff was used since visible skin damage was not detected in stimulated paws within 24 h. Stimulation for 18 sec, on the other hand, resulted in visible skin damage. Generally, latency to paw withdrawal is ~8 sec with our C stimulus parameters.

### Behavioral testing

The testing paradigm is similar to an earlier protocol that we established using a radiant heat stimulus from a focused incandescent light source [54]. Rats were placed unrestrained under plastic enclosures on an elevated glass platform. The enclosures (23 × 13 × 13 cm) were large enough for the rats to move freely. Rats required 5-10 min to habituate. The laser collimator was attached to a support and positioned below the glass, with the beam perpendicular to the surface. Great care was taken to keep the glass surface dry and free of debris or excrement.

The endpoint for C-fiber response is paw withdrawal latency. In this case, when the rat was still with paw flat on glass, the beam was aimed at the mid-plantar foot pad then fired continuously until withdrawal. The latency to withdrawal was measured by the experimenter using a digital stopwatch. Preliminary studies determined that typical basal withdrawal latencies for adult Sprague-Dawley rats (250-350 g) subjected to a 0.083 W/mm^2 ^stimulus was between 8-9 sec, similar to the latency obtained with a white-light radiant heat stimulus [54].

The response to a high-energy Aδ stimulus in untreated, normal rats is brisk and behaviorally productive, characterized by rapid paw withdrawal, orientation of the head to the stimulated paw, and paw shaking and licking, which is similar to that observed when using a CO_2 _laser [28,55]. Since withdrawal latency was generally too rapid to be an informative endpoint for the Aδ response, we instead used a binary measure (no withdrawal = 0, withdrawal = 1), and calculated the probability of withdrawal to each stimulus intensity (total withdrawals/total number of trials). Rats, 3 to 4 at a time, were placed on an elevated glass test platform and stimulated sequentially; the inter-stimulus interval, per rat, was typically ~1 min. Two different stimuli were used where the lower stimulus intensity (5.12 W/mm^2^) always preceded testing of the higher intensity (6.08 W/mm^2^). Mid-plantar site was stimulated on each rat and then the toe on each rat. On any one test day, two different spots on the mid-plantar region were stimulated, one with 5.12 and the other with 6.08 W/mm^2^; also two different toes were stimulated: the index toe received 5.12 W/mm^2 ^and the middle toe received 6.08 W/mm^2^, for a total of 4 stimuli per rat per day unless noted otherwise. Responses to C-fiber stimuli (mid-plantar) were determined at least 10 min after evaluation of Aδ responses. Only one C-fiber test was administered per day. When C-fiber responses were tested on toes, the same spot diameter of 5 mm was used. The toes in these rats are at least 5 mm long but 2 mm wide. The stimulus was applied in such a way as to ensure that an area of 5 × 2 mm was heated. Aδ responses were not determined on days when the toes were stimulated with the C-fiber stimulus. To assess the effects of RTX, responses from each animal (n = 6) were grouped according to stimulation site (mid-plantar or toe) and stimulus intensity (5.12 or 6.08 W/mm^2^). Responses from 2 or three test days during the indicated time period (weeks) were pooled and averaged to obtain mean response values. To determine statistical significance, repeated measures 2-way ANOVA was used where responses were compared between vehicle- and RTX-treated paws in each animal.

A subjective rating scale for behavioral intensity was also developed to compare the effect of RTX on withdrawal responses. There was nearly complete concurrence on the rating in all tests when performed independently (JMK, KM, MJI or BDB). The categories were: 0 = no visible response; 1 = slight twitch of the body or abrupt movement of head; 2 = withdrawal of the foot off the glass, including either a rapid return of the paw to the glass as well as the rat walking away; 3 = withdrawal of the foot characterized by prolonged paw shaking or guarding, and orientation of the head, without licking; 4 = strong withdrawal, which includes paw shaking, orientation and licking. Although this endpoint was useful in characterizing the degree of the nocifensive behavior associated with Aδ stimulation, we cannot say for certain whether licking, which sometimes occurred well after paw withdrawal, is mediated by Aδ-fibers, C-fibers or both.

### High-Speed Videography

A high-speed 12-bit monochrome camera (AOS Technologies AG, Switzerland) was used to precisely capture rapid movements associated with withdrawal activity. Images were acquired at 500 frames per second and recorded for a total of 2 sec, which was sufficient to capture the entire 100 ms laser pulse (IR light appears as a saturating flash for 50 frames) and withdrawal of the stimulated limb. Rats used for recording were initially conditioned to the test for 3 days prior (not consecutive). Many preliminary recordings (> 40) of stimulated rats were taken to determine the best conditions, by varying such factors as the viewing angle and illumination. Although ultimately not all were used for the data in Table 1 they were constructive to developing our hypothesis. In some recordings, to better view the musculature, fur on rats hind quarters was shaved off to reveal the lower back, hips, and legs. Great care was taken to only record when the rats were properly habituated and sitting still at the moment of stimulation, irrespective of posture. For the data presented in Table 1, 4 rats total were used, and the middle or index toe was targeted with the 100 ms, 6.08 W/mm^2 ^stimulus. On each day (2 total) of video recording, only one stimulation was performed on both the left and right toes, totaling 8 trials/day. The index toe was used on one day while the middle was used for the second. For calculating latency to withdrawal, frames were counted from the onset of laser stimulation to the onset of the first-observable movement or muscle twitch. In separate experiments, these rats were also stimulated at the same laser intensity but with progressively shorter pulse durations (increments of 10 ms) to determine the minimum time required to initiate a response. Stimulation for 30 ms did not evoke withdrawal responses (0/8) whereas stimulation for 40 ms evoked withdrawal in 25% of the trials (2/8). Thus, when determining the latency, 30 ms was subtracted from the total time, since the fibers were not sufficiently heated by 30 ms to initiate a response. Therefore the noxious phase of the 6.08 W/mm^2 ^stimulus was defined as the last 70 of 100 ms. An estimation of the conduction velocity was then calculated using the length of the exposed nerve, by gross dissection, from the toe to the lamina 5 dorsal root entry zone.

### Immunofluorescence and cell counting

Immunohistochemistry was performed on 12 μm fresh frozen sections, which were immersion fixed for 10 min in 4% buffered formaldehyde at room temperature and subjected to antigen retrieval by boiling (20 min) in target retrieval solution, high pH (DAKO, Glostrup, Denmark). The primary antibody was a rabbit anti-rat c-*fos *[56] diluted 1:10 K. For immunofluorescence staining, secondary antibodies conjugated with Alexa Fluor 488 (Invitrogen) was used. Images were obtained with an epifluorescence microscope attached to CCD camera (Olympus) using Olympus software to take images of all samples under the same exposure time. For cell counting, an immunofluorescence intensity cut-off was established to eliminate counting of basally expressed c-Fos in the medial portion of the spinal cord.

### RT-PCR Analysis

RTX (50 ng) was injected into one paw and vehicle was injected into the contralateral paw. Animals were sacrificed 6, 24, 120 or 240 h later, dorsal root ganglia were removed and RNA was extracted as previously described [57]. RT-PCR was performed using the Access RT-PCR system (Promega, Madison, WI). The PCR primer pairs are 5'-CTGTGGTTTTTGGTGGGAAG-3' and 5'-GGCCATGTAAACTGGCTGAT-3' for ATF-3 (250 bp); 5'-CCAGAAACCAGCCAACTCTC-3'and 5'-CCGACTCATTGGGATCATCT-3' for MCP-1 (192 bp); 5'-ACCACAGTCCATGCCATCAC-3' and 5'-TCCACCACCCTGTTGCTGTA-3' for GPDH (452 bp). The RT-PCR analysis was performed according to the manufacturer's instruction in 25 μL reaction mixture containing exactly 8 ng of RNA. RT-PCR steps were 1 cycle of 45 min at 45°C for reverse transcription, 1 cycle of 2 min at 94°C for inactivation of transcriptase, 26-30 cycles of 30 s at 94°C for denaturation, 1 min at 55°C for annealing, 2 min at 68°C for extension, and final extension at 68°C for 7 min. The RT-PCR products were separated by electrophoresis on 2% agarose/ethidium bromide gels and images were acquired with an AlphaImager system (Alpha Innotech Corp.). The relative intensities of the RT-PCR products, as visualized on the gel, were analyzed quantitatively using ImageQuant 5 software. The results were normalized to GPDH. Comparisons of gene expression from ipsilateral and contralateral tissues were made by paired Student's t-test.

## Competing interests

MIN is the CEO of Lasmed, LLC., which provided the laser and initial stimulus protocol for this work.

## Authors' contributions

BDB, JN, ML carried out the behavioral studies. JN, ML, NM and LS participated in the immunohistochemical and gene analysis. KM, BDB, JMK and MJI participated in the design of the study and performed data analysis. KM, JMK, MIN and MJI coordinated the study and drafted the manuscript. GH conceived of and conducted the high-speed videography recordings. All authors read and approved the final manuscript.

## Supplementary Material

Additional file 1 **Aδ laser stimulation of rat hind paw captured with high-speed videography**. The rat was recorded at a rate of 500 frames per sec (fps) to capture the first-observable movement after laser stimulation (6.08 W/mm^2^). The laser stimulus (100 ms, 31 frames) can be easily observed since infrared light saturates the CCD sensor, appearing as black pixels in the video. The rat was at rest and motionless immediately before stimulation. Post-stimulation, ostensible movement could be detected in the left forearm and the right shoulder by 104 ms. Clear movement of the contralateral paw could be detected by 4 frames (113 ms). Complete limb withdrawal and paw licking occur several frames later. The total length of the recording is 1 sec. Withdrawal of the stimulated limb appears rapid and very brisk when viewed in real-time. [Note: Due to video compression, this reformatted video file plays at ~310 fps.] Click here for file

Additional file 2 **Upregulation of galanin following intraplantar injection of RTX (50 ng)**. Gel image shows ganglionic expression levels of mRNA encoding galanin 5 or 10 days (**A **and **B**, respectively) after vehicle or RTX treatment, taken from left and right L4-L5 dorsal root ganglia (n = 4 rats, ipsilateral RTX expression is denoted by an asterisk). Graph in (**C**) shows that galanin transcript levels (after GPDH normalization) were significantly altered by intraplantar RTX injection. Graph is presented as mean ± SEM. (N = 4/group). ***P < 0.001 as determined by one-way ANOVA followed by a Bonferroni correction. ATF3 transcript levels were also increased on day 5 whereas MCP-1 levels were not (data not shown). Click here for file

## References

[B1] Caterina MJ Leffler A Malmberg AB Martin WJ Trafton J Petersen-Zeitz KR Koltzenburg M Basbaum AI Julius D Impaired nociception and pain sensation in mice lacking the capsaicin receptor Science 2000 288 306 313 10.1126/science.288.5464.306 10764638

[B2] Davis JB Gray J Gunthorpe MJ Hatcher JP Davey PT Overend P Harries MH Latcham J Clapham C Atkinson K Hughes SA Rance K Grau E Harper AJ Pugh PL Rogers DC Bingham S Randall A Sheardown SA Vanilloid receptor-1 is essential for inflammatory thermal hyperalgesia Nature 2000 405 183 187 10.1038/35012076 10821274

[B3] Caterina MJ Schumacher MA Tominaga M Rosen TA Levine JD Julius D The capsaicin receptor: a heat-activated ion channel in the pain pathway Nature 1997 389 816 824 10.1038/39807 9349813

[B4] Belmonte C Gallar J Pozo MA Rebollo I Excitation by irritant chemical substances of sensory afferent units in the cat's cornea J Physiol 1991 437 709 725 1890657 10.1113/jphysiol.1991.sp018621 PMC1180073

[B5] Kaufman MP Iwamoto GA Longhurst JC Mitchell JH Effects of capsaicin and bradykinin on afferent fibers with ending in skeletal muscle Circ Res 1982 50 133 139 7053873 10.1161/01.res.50.1.133

[B6] Yeomans DC Proudfit HK Nociceptive responses to high and low rates of noxious cutaneous heating are mediated by different nociceptors in the rat: electrophysiological evidence Pain 1996 68 141 150 10.1016/S0304-3959(96)03177-6 9252009

[B7] Voight EA Kort ME Transient receptor potential vanilloid-1 antagonists: a survey of recent patent literature Expert Opin Ther Pat 2010 20 1107 1122 10.1517/13543776.2010.497756 20586701

[B8] Wong GY Gavva NR Therapeutic potential of vanilloid receptor TRPV1 agonists and antagonists as analgesics: Recent advances and setbacks Brain Res Rev 2009 60 267 277 10.1016/j.brainresrev.2008.12.006 19150372

[B9] Szallasi A Cortright DN Blum CA Eid SR The vanilloid receptor TRPV1: 10 years from channel cloning to antagonist proof-of-concept Nat Rev Drug Discov 2007 6 357 372 10.1038/nrd2280 17464295

[B10] Yaksh TL Farb DH Leeman SE Jessell TM Intrathecal capsaicin depletes substance P in the rat spinal cord and produces prolonged thermal analgesia Science 1979 206 481 483 10.1126/science.228392 228392

[B11] Szallasi A Blumberg PM Vanilloid receptors: new insights enhance potential as a therapeutic target Pain 1996 68 195 208 10.1016/S0304-3959(96)03202-2 9121806

[B12] Karai LJ Russell JT Iadarola MJ Olah Z Vanilloid receptor 1 regulates multiple calcium compartments and contributes to Ca2+-induced Ca2+ release in sensory neurons J Biol Chem 2004 279 16377 16387 10.1074/jbc.M310891200 14963041

[B13] Winter J Dray A Wood JN Yeats JC Bevan S Cellular mechanism of action of resiniferatoxin: a potent sensory neuron excitotoxin Brain Res 1990 520 131 140 10.1016/0006-8993(90)91698-G 2169951

[B14] Karai L Brown DC Mannes AJ Connelly ST Brown J Gandal M Wellisch OM Neubert JK Olah Z Iadarola MJ Deletion of vanilloid receptor 1-expressing primary afferent neurons for pain control J Clin Invest 2004 113 1344 1352 15124026 10.1172/JCI20449 PMC398431

[B15] Brown DC Iadarola MJ Perkowski SZ Erin H Shofer F Laszlo KJ Olah Z Mannes AJ Physiologic and antinociceptive effects of intrathecal resiniferatoxin in a canine bone cancer model Anesthesiology 2005 103 1052 1059 10.1097/00000542-200511000-00020 16249680

[B16] Jeffry JA Yu SQ Sikand P Parihar A Evans MS Premkumar LS Selective targeting of TRPV1 expressing sensory nerve terminals in the spinal cord for long lasting analgesia PLoS One 2009 4 e7021 10.1371/journal.pone.0007021 19753113 PMC2737142

[B17] Neubert JK Karai L Jun JH Kim HS Olah Z Iadarola MJ Peripherally induced resiniferatoxin analgesia Pain 2003 104 219 228 10.1016/S0304-3959(03)00009-5 12855332

[B18] Bates BD Mitchell K Keller JM Chan CC Swaim WD Yaskovich R Mannes AJ Iadarola MJ Prolonged analgesic response of cornea to topical resiniferatoxin, a potent TRPV1 agonist Pain 2010 149 522 528 10.1016/j.pain.2010.03.024 20403666 PMC2913152

[B19] Szolcsanyi J Szallasi A Szallasi Z Joo F Blumberg PM Resiniferatoxin. An ultrapotent neurotoxin of capsaicin-sensitive primary afferent neurons Ann N Y Acad Sci 1991 632 473 475 10.1111/j.1749-6632.1991.tb33161.x 1952635

[B20] Cavanaugh DJ Lee H Lo L Shields SD Zylka MJ Basbaum AI Anderson DJ Distinct subsets of unmyelinated primary sensory fibers mediate behavioral responses to noxious thermal and mechanical stimuli Proc Natl Acad Sci USA 2009 106 9075 9080 10.1073/pnas.0901507106 19451647 PMC2683885

[B21] Mishra SK Hoon MA Ablation of TrpV1 neurons reveals their selective role in thermal pain sensation Mol Cell Neurosci 2010 43 157 163 10.1016/j.mcn.2009.10.006 19853036 PMC2818468

[B22] Kalliomaki J Weng HR Nilsson HJ Schouenborg J Nociceptive C fibre input to the primary somatosensory cortex (SI). A field potential study in the rat Brain Res 1993 622 262 270 10.1016/0006-8993(93)90827-A 8242365

[B23] Greffrath W Nemenov MI Schwarz S Baumgartner U Vogel H Arendt-Nielsen L Treede RD Inward currents in primary nociceptive neurons of the rat and pain sensations in humans elicited by infrared diode laser pulses Pain 2002 99 145 155 10.1016/S0304-3959(02)00071-4 12237192

[B24] Tzabazis A Klyukinov M Manering N Nemenov MI Shafer SL Yeomans DC Differential activation of trigeminal C or Adelta nociceptors by infrared diode laser in rats: behavioral evidence Brain Res 2005 1037 148 156 10.1016/j.brainres.2005.01.019 15777763

[B25] Cuellar JM Manering NA Klukinov M Nemenov MI Yeomans DC Thermal nociceptive properties of trigeminal afferent neurons in rats Mol Pain 2010 6 39 10.1186/1744-8069-6-39 20609212 PMC2910000

[B26] Sun JJ Yang JW Shyu BC Current source density analysis of laser heat-evoked intra-cortical field potentials in the primary somatosensory cortex of rats Neuroscience 2006 140 1321 1336 10.1016/j.neuroscience.2006.03.018 16675140

[B27] Devor M Carmon A Frostig R Primary afferent and spinal sensory neurons that respond to brief pulses of intense infrared laser radiation: a preliminary survey in rats Exp Neurol 1982 76 483 494 10.1016/0014-4886(82)90118-2 7084369

[B28] Fan RJ Kung JC Olausson BA Shyu BC Nocifensive behaviors components evoked by brief laser pulses are mediated by C fibers Physiol Behav 2009 98 108 117 10.1016/j.physbeh.2009.04.022 19410593

[B29] Lawson SN Perry MJ Prabhakar E McCarthy PW Primary sensory neurones: neurofilament, neuropeptides, and conduction velocity Brain Res Bull 1993 30 239 243 10.1016/0361-9230(93)90250-F 7681350

[B30] Harper AA Lawson SN Conduction velocity is related to morphological cell type in rat dorsal root ganglion neurones J Physiol 1985 359 31 46 3999040 10.1113/jphysiol.1985.sp015573 PMC1193363

[B31] Lawson JJ McIlwrath SL Woodbury CJ Davis BM Koerber HR TRPV1 unlike TRPV2 is restricted to a subset of mechanically insensitive cutaneous nociceptors responding to heat J Pain 2008 9 298 308 10.1016/j.jpain.2007.12.001 18226966 PMC2372162

[B32] Danneman PJ Kiritsy-Roy JA Morrow TJ Casey KL Central delay of the laser-activated rat tail-flick reflex Pain 1994 58 39 44 10.1016/0304-3959(94)90183-X 7970838

[B33] Bakels R Kernell D Matching between motoneurone and muscle unit properties in rat medial gastrocnemius J Physiol 1993 463 307 324 8246185 10.1113/jphysiol.1993.sp019596 PMC1175345

[B34] Gosselin RD Dansereau MA Pohl M Kitabgi P Beaudet N Sarret P Melik Parsadaniantz S Chemokine network in the nervous system: a new target for pain relief Curr Med Chem 2008 15 2866 2875 10.2174/092986708786242822 18991641

[B35] Jung H Toth PT White FA Miller RJ Monocyte chemoattractant protein-1 functions as a neuromodulator in dorsal root ganglia neurons J Neurochem 2008 104 254 263 17944871 10.1111/j.1471-4159.2007.04969.x PMC2186066

[B36] Yang HY Mitchell K Keller JM Iadarola MJ Peripheral inflammation increases Scya2 expression in sensory ganglia and cytokine and endothelial related gene expression in inflamed tissue J Neurochem 2007 103 1628 1643 10.1111/j.1471-4159.2007.04874.x 17883394

[B37] Tsujino H Kondo E Fukuoka T Dai Y Tokunaga A Miki K Yonenobu K Ochi T Noguchi K Activating transcription factor 3 (ATF3) induction by axotomy in sensory and motoneurons: A novel neuronal marker of nerve injury Mol Cell Neurosci 2000 15 170 182 10.1006/mcne.1999.0814 10673325

[B38] Noguchi K Kowalski K Traub R Solodkin A Iadarola MJ Ruda MA Dynorphin expression and Fos-like immunoreactivity following inflammation induced hyperalgesia are colocalized in spinal cord neurons Brain Res Mol Brain Res 1991 10 227 233 10.1016/0169-328X(91)90065-6 1679515

[B39] Draisci G Iadarola MJ Temporal analysis of increases in c-fos, preprodynorphin and preproenkephalin mRNAs in rat spinal cord Brain Res Mol Brain Res 1989 6 31 37 10.1016/0169-328X(89)90025-9 2570339

[B40] Qiao ZM Wang JY Han JS Luo F Dynamic processing of nociception in cortical network in conscious rats: a laser-evoked field potential study Cell Mol Neurobiol 2008 28 671 687 10.1007/s10571-007-9216-3 17922183 PMC11515030

[B41] Isseroff RG Sarne Y Carmon A Isseroff A Cortical potentials evoked by innocuous tactile and noxious thermal stimulation in the rat: differences in localization and latency Behav Neural Biol 1982 35 294 307 10.1016/S0163-1047(82)90725-7 7181820

[B42] Price DD Hu JW Dubner R Gracely RH Peripheral suppression of first pain and central summation of second pain evoked by noxious heat pulses Pain 1977 3 57 68 10.1016/0304-3959(77)90035-5 876667

[B43] Plaghki L Decruynaere C Van Dooren P Le Bars D The fine tuning of pain thresholds: a sophisticated double alarm system PLoS One 2010 5 e10269 10.1371/journal.pone.0010269 20428245 PMC2859063

[B44] McIlroy WE Bent LR Potvin JR Brooke JD Maki BE Preparatory balance adjustments precede withdrawal response to noxious stimulation in standing humans Neurosci Lett 1999 267 197 200 10.1016/S0304-3940(99)00365-1 10381010

[B45] Bent LR Potvin JR Brooke JD McIlroy WE Medio-lateral balance adjustments preceding reflexive limb withdrawal are modified by postural demands Brain Res 2001 914 100 105 10.1016/S0006-8993(01)02782-2 11578602

[B46] Beydoun A Dyke DB Morrow TJ Casey KL Topical capsaicin selectively attenuates heat pain and A delta fiber-mediated laser-evoked potentials Pain 1996 65 189 196 10.1016/0304-3959(95)00161-1 8826506

[B47] Rage M Van Acker N Facer P Shenoy R Knaapen MW Timmers M Streffer J Anand P Meert T Plaghki L The time course of CO2 laser-evoked responses and of skin nerve fibre markers after topical capsaicin in human volunteers Clin Neurophysiol 2010 121 1256 1266 10.1016/j.clinph.2010.02.159 20347388

[B48] Bromm B Neitzel H Tecklenburg A Treede RD Evoked cerebral potential correlates of C-fibre activity in man Neurosci Lett 1983 43 109 114 10.1016/0304-3940(83)90137-4 6669318

[B49] Veldhuijzen DS Nemenov MI Keaser M Zhuo J Gullapalli RP Greenspan JD Differential brain activation associated with laser-evoked burning and pricking pain: An event-related fMRI study Pain 2009 141 104 113 10.1016/j.pain.2008.10.027 19058914 PMC6449044

[B50] White FA Sun J Waters SM Ma C Ren D Ripsch M Steflik J Cortright DN Lamotte RH Miller RJ Excitatory monocyte chemoattractant protein-1 signaling is up-regulated in sensory neurons after chronic compression of the dorsal root ganglion Proc Natl Acad Sci USA 2005 102 14092 14097 10.1073/pnas.0503496102 16174730 PMC1236537

[B51] Bhangoo S Ren D Miller RJ Henry KJ Lineswala J Hamdouchi C Li B Monahan PE Chan DM Ripsch MS White FA Delayed functional expression of neuronal chemokine receptors following focal nerve demyelination in the rat: a mechanism for the development of chronic sensitization of peripheral nociceptors Mol Pain 2007 3 38 10.1186/1744-8069-3-38 18076762 PMC2228278

[B52] Jeon SM Lee KM Cho HJ Expression of monocyte chemoattractant protein-1 in rat dorsal root ganglia and spinal cord in experimental models of neuropathic pain Brain Res 2009 1251 103 111 10.1016/j.brainres.2008.11.046 19059387

[B53] Seijffers R Allchorne AJ Woolf CJ The transcription factor ATF-3 promotes neurite outgrowth Mol Cell Neurosci 2006 32 143 154 10.1016/j.mcn.2006.03.005 16713293

[B54] Iadarola MJ Brady LS Draisci G Dubner R Enhancement of dynorphin gene expression in spinal cord following experimental inflammation: stimulus specificity, behavioral parameters and opioid receptor binding Pain 1988 35 313 326 10.1016/0304-3959(88)90141-8 2906426

[B55] Fan RJ Shyu BC Hsiao S Analysis of nocifensive behavior induced in rats by CO2 laser pulse stimulation Physiol Behav 1995 57 1131 1137 10.1016/0031-9384(94)00372-C 7652034

[B56] Young ST Porrino LJ Iadarola MJ Cocaine induces striatal c-fos-immunoreactive proteins via dopaminergic D1 receptors Proc Natl Acad Sci USA 1991 88 1291 1295 10.1073/pnas.88.4.1291 1825356 PMC51003

[B57] Mitchell K Iadarola MJ RT-PCR analysis of pain genes: use of gel-based RT-PCR for studying induced and tissue-enriched gene expression Methods Mol Biol 2010 617 279 295 full_text 20336429 10.1007/978-1-60327-323-7_21 PMC3417750

