# Supplementary figures and images for: Ablation of rat TRPV1-expressing Adelta/C-fibers with resiniferatoxin: analysis of withdrawal behaviors, recovery of function and molecular correlates
Source: Mol Pain. 2010 Dec 17;6:94. doi: 10.1186/1744-8069-6-94 (PMC3019206; doi:10.1186/1744-8069-6-94)

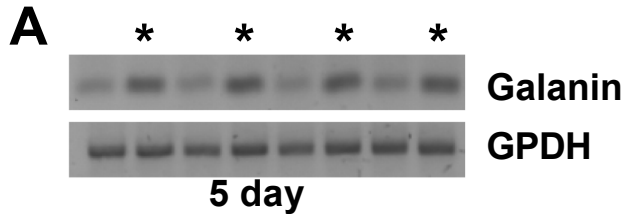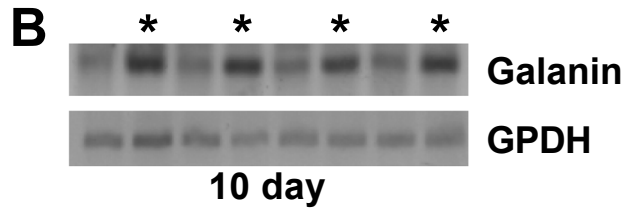

**C**

## Galanin Expression

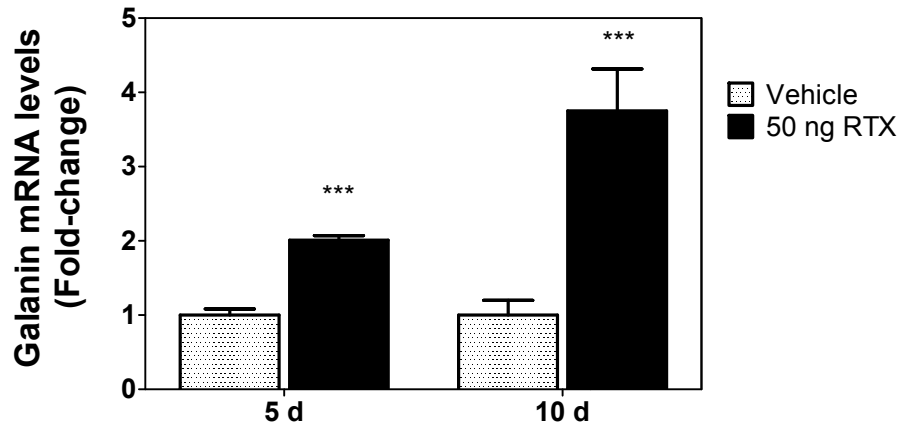

Supplement: Additional file 2 — Upregulation of galanin following intraplantar injection of RTX (50 ng). Gel image shows ganglionic expression levels of mRNA encoding galanin 5 or 10 days (A and B, respectively) after vehicle or RTX treatment, taken from left and right L4-L5 dorsal root ganglia (n = 4 rats, ipsilateral RTX expression is denoted by an asterisk). Graph in (C) shows that galanin transcript levels (after GPDH normalization) were significantly altered by intraplantar RTX injection. Graph is presented as mean ± SEM. (N = 4/group). ***P < 0.001 as determined by one-way ANOVA followed by a Bonferroni correction. ATF3 transcript levels were also increased on day 5 whereas MCP-1 levels were not (data not shown). [file 1744-8069-6-94-S2.PDF]
